# Immunoaffinity proteomics for kidney injury biomarkers in male beagle dogs

**DOI:** 10.17179/excli2023-6621

**Published:** 2024-01-15

**Authors:** Wael Naboulsi, Hannes Planatscher, Felix F. Schmidt, Andreas Steinhilber, Thomas O. Joos, Adeyemi O. Adedeji, J. Eric McDuffie, Oliver Poetz

**Affiliations:** 1 Signatope GmbH, Reutlingen, Germany; 2 NMI Natural and Medical Sciences Institute at the University of Tuebingen, Reutlingen, Germany; 3 Department of Safety Assessment, Genentech, A Member of the Roche Group, South San Francisco, CA, USA; 4 Janssen Pharmaceutical Research & Development LLC, San Diego, California, USA; 5 Neurocrine Biosciences, Inc., San Diego, California, USA

**Keywords:** drug-induced kidney injury, biomarkers, proteomics, immunoaffinity-LC-MS

## Abstract

Drug-induced kidney injury (DIKI) is a cause of drug development failure. Dogs represent a common non-rodent animal model in pre-clinical safety studies; however, biomarker assays for detecting nephrotoxicity in dogs are limited. To identify novel proteins and gain insight into the molecular mechanisms involved in DIKI, we developed an assay to evaluate proteomic changes associated with DIKI in male beagle dogs that received nephrotoxic doses of tobramycin for 10 consecutive days. Label-free quantitative discovery proteomics analysis on representative kidney cortex tissues collected on Day 11 showed that the tobramycin-induced kidney injury led to a significant differential regulation of 94 proteins mostly associated with mechanisms of nephrotoxicity such as oxidative stress and proteasome degradation. For verification of the proteomic results, we developed a multiplex peptide-centric immunoaffinity liquid chromatography tandem mass spectrometry assay (IA LC-MS/MS) to evaluate the association of eight DIKI protein biomarker candidates using kidney cortices collected on Day 11 and urine samples collected on Days -4, 1, 3, 7 and 10. The results showed that most biomarkers evaluated were detected in the kidney cortices and their expression profile in tissue aligned with the label-free data. Cystatin C was the most consistent marker regardless of the magnitude of the renal injury while fatty acid-binding protein-4 (FABP4) and kidney injury molecule-1 (KIM-1) were the most affected biomarkers in response to moderate proximal tubular injury in absence of changes in serum-based concentrations of blood urea nitrogen or creatinine. In the urine, clusterin is considered the most consistent biomarker regardless of the magnitude and time of the renal injury. To our knowledge, this is the most comprehensive multiplex assay for the quantitative analysis of mechanism-based proximal tubular injury biomarkers in dogs.

## Introduction

Balancing both the risk of toxicity and the efficacy of treatment for patients is the primary objective of preclinical and clinical research during the drug development process. One-third of drug candidates fail to meet drug safety requirements and drug-induced kidney injury (DIKI) accounts for 9 % of such occurrences (Cook et al., 2014[7]; Obajdin et al., 2018[30]). The most efficient way to minimize or prevent drug-induced nephrotoxicity in humans is to implement sensitive tools that can detect drug toxicity during the early stages of preclinical drug development (Vaidya et al., 2008[36]). This can be achieved by deploying translational biomarkers in routine preclinical animal studies. Such translational biomarker analyses can detect potential kidney injury and subsequently facilitate the prevention of nephrotoxicity during clinical trials.

Blood urea nitrogen (BUN) and serum creatinine (sCr) are routinely used as blood-based biomarkers of kidney injury in clinical and preclinical studies. However, both BUN and sCr demonstrate low sensitivity and specificity and mainly reflect functional changes in the glomerular filtration rate (GFR) (Waikar and Bonventre 2008[39]; Yu et al., 2010[42]). Extensive preclinical research has focused on identifying and qualifying renal injury biomarkers to overcome these limitations (Ennulat and Adler 2015[12]). In 2018, a composite measure of six urinary (UR) biomarkers: clusterin (UR CLU), cystatin-C (UR CST3), kidney injury molecule-1 (UR KIM-1), N-acetyl-beta-D-glucosaminidase (NAG), neutrophil gelatinase-associated lipocalin (UR NGAL), and osteopontin (UR OPN) - was considered by the Predictive Safety Testing Consortium to be suitable for detecting kidney tubular injury in phase 1 trials, in conjunction with conventional measures (FDA, 2018[13]). Additionally, UR CST3 as well as urinary alpha-1-microglobulin also known as bikunin precursor (UR AMBP) and total protein (UR PROT) were discussed as potential biomarkers for glomerular injury and/or impaired tubular reabsorption (Gautier et al., 2016[15]; Liborio et al., 2014[25]). 

Having a translatable biomarker which can be implemented across species boundaries is of particular interest due to species-specific differences in nephrotoxicity (Bonventre et al., 2010[4]; Mutlib et al., 2000[29]). This emphasizes the need to investigate the biomarker candidates in non-rodent preclinical animal studies to endorse their application as translatable biomarkers. In this regard, the performance of some of the novel urinary renal tubular safety biomarkers (i.e., UR CLU, UR NGAL, UR CST3, UR OPN, UR NAG, albumin (UR ALB) and UR PROT) was well demonstrated in dogs (Gu et al., 2018[17]; McDuffie et al., 2016[27]). Additionally, retinol binding protein (RBP4) was investigated as novel urinary renal tubular safety biomarkers in cynomolgus monkeys (Gu et al., 2018[17]). Yet, due to the lack of dog immunoassays for some of these proteins, an investigation of all biomarker candidates was not feasible. In addition, there is no single dog multiplex analytical method available that covers the range of the potential protein biomarker candidates. This underscores the urgent need to develop new assays that could bridge the analytical gap between humans and dogs as well as other non-rodent species. In the literature on nephrotoxicity, there is abundant evidence indicating that epithelial cells die either by necrosis or apoptosis (Gandhi et al., 2014[14]). However, detailed data describing the mechanisms of nephrotoxicity in dogs at the proteome level are currently lacking.

In this study, we conducted a label-free proteome analysis on kidney cortex tissue samples of male beagle dogs subjected to tubular region-specific injury induced by tobramycin; yet, in absence of changes in the concentrations of BUN or sCr. The proximal tubular injury was confirmed by microscopic evaluation. Gene ontology analysis of differentially expressed proteins in kidney tissues revealed protein molecular changes associated with oxidative stress and proteasome degradation in dogs with DIKI. Subsequently, a targeted immunoaffinity liquid chromatography tandem mass spectrometry assay (IA-LC-MS/MS) was developed to evaluate the potential application of a panel of established and novel biomarkers detected in representative dog kidney cortex tissue and urine. Overall, the combination of these results will help scientists gain insights into the molecular mechanisms associated with DIKI in dogs while using the renal biomarkers to support early monitoring for proximal tubular injury during preclinical toxicology studies.

## Materials and Methods

### Ethical statement

All procedures were conducted according to a protocol approved by the Institutional Animal Care and Use Committee at a facility accredited by the Association for Assessment and Accreditation of Laboratory Animal Care International, which complies with the current recommendations of the Guide for the Care and Use of Laboratory Animals (National Research Council (U.S.), Committee for the Update of the Guide for the Care and Use of Laboratory Animals, Institute for Laboratory Animal Research (U.S.) & National Academies Press (U.S.), 2011). This work was conducted in accordance with the 3Rs (replacement, reduction, and refinement) principles. Therefore, the adult male beagle dogs were first deployed for evaluating toxicokinetic parameters after single low-dose administration of proprietary compounds which were structurally unrelated to tobramycin. All dogs underwent an extensive wash-out period lasting at least one month, prior to the animal pre-selection phase of this study. Repurposed animal selection criteria included baseline (pre-study) levels of clinical pathology (i.e., serum and urine chemistry) parameter profiles that were within the reference range for the facility, indicating ongoing organ injury(s) was either absent or not discernable before combining routine histopathology and/or more sensitive and selective safety biomarkers.

### Animals and husbandry

**Figure 1:**
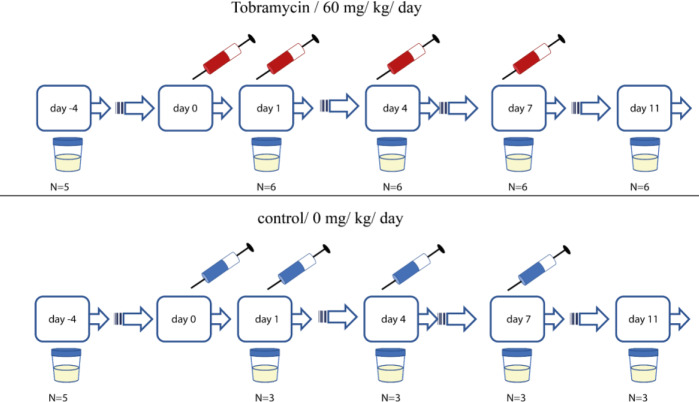
Sample collection scheme for male beagle dogs after once daily administration of tobramycin (60 mg/kg/day; n=6 or vehicle (saline; n=3) by intramuscular injection for 10 consecutive days. Representative urine and blood samples were collected at pre-treatment (Day -4) and pre-dosing (Days 1, 3, 7 and 10) and at necropsy (Day 11). Representative kidney tissue samples were collected at necropsy (Day 11).

Eleven male beagle dogs of approximately one year of age were purchased from Marshall Bioresources, North Rose, NY, USA. Water, food, and enriched nutrition were provided by reverse osmosis for ad libitum consumption via bowls, except where noted. Animals were provided a 1:1 mix of adult dog dry food and adult dog canned wet food (approximately 125 g) each for ad libitum consumption via a food bowl. Enrichment was provided once daily in the afternoon (at approximately 1.00 p.m.). During a two-week acclimatization period, nine dogs were randomly assigned to the study and were subsequently housed individually in metabolic cages. For baseline biomarker analysis, urine samples were collected overnight on wet ice via pan catch, during a 16-hour (h) fasting period at pre-dose (on Day -4). Dogs received nephrotoxic doses of tobramycin (60 mg/kg/ day, n=6) or vehicle (saline; n=3; n=3) for 10 consecutive days. Urine samples were collected overnight on wet ice via pan catch during a 16-h fasting period at post-dose on Day 1, Day 4, Day 9, and Day 10. The treatment regime and urine, blood and tissue sample collection time points are depicted in Figure 1(Fig. 1). The blood-based data sets are not shown given a lack of adverse changes in clinical pathology parameters, for example, BUN or CR in serum. During the fasting period, all animals had access to water *ad libitum* via water bowls. Total urine volumes (Ur Vol) were recorded and immediately placed on wet ice for up to 1 h prior to centrifugation at 800 x g for at least 10 min at 4 °C. Urine supernatants were prepared as aliquots and stored at -80 °C, until time of analysis.

### Pathology

**Figure 2:**
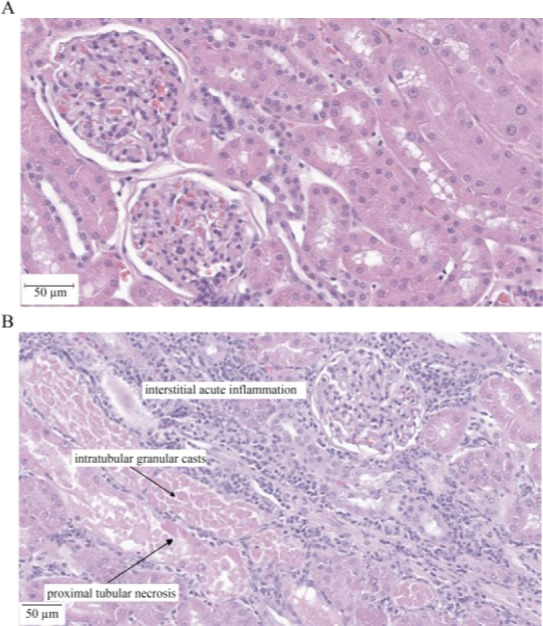
Representative hematoxylin and eosin-stained, formalin-fixed, paraffin-embedded kidneys from male beagle dogs following once daily administration of tobramycin (60 mg/kg/day; n=6) or vehicle (saline; n=3) by intramuscular injection for 10 consecutive days; and necropsy on Day 11. There was no injury identified in any vehicle group animals (A). In the representative tobramycin-treated dog presented, microscopic findings included bilateral, multifocal, moderate proximal tubular necrosis, moderate intratubular granular casts, and slight/mild interstitial acute inflammation [data shown] (B); and slight/mild hyaline casts were also observed [data not shown].

Dogs were euthanized with an intramuscularly administered overdose of barbiturate as per the contract research laboratory's standard operating procedures on study Day 11 after terminal blood and urine collections. All animals were necropsied and both the left and the right kidneys were examined for gross lesions. Four representative pieces of both the left and the right kidneys were cut transversely or longitudinally including cortex, medulla, and papilla, collected and snap frozen in separate vials using liquid nitrogen, while the remaining right and left kidney tissues were fixed in 10 % neutral buffered formalin for a minimum of 24 h, processed, embedded in paraffin, sectioned at 4-5 μm, and stained with hematoxylin and eosin (H&E). Histopathological characterization kidney injury was performed by an anatomic pathologist that was board-certified by the American College of Veterinary Pathologists. Histologic slide evaluations were conducted in a blinded fashion using a semi-quantitative scale in which the percentages of proximal cortical tubules showing necrosis, intratubular cast, hyaline cast(s), or acute inflammation were assigned a score: Grade 1 = minimal; Grade 2 = slight/mild; Grade 3 = moderate; and Grade 4 = marked. Representative photomicrographs of H&E-stained kidney sections (Figure 2(Fig. 2)) were generated using the Leica Aperio AT2 scanner (Deer Park, IL, USA).

### Sample preparation and protein digestion

Tissue samples were lysed and homogenized in sample lysis buffer (sodium dihydrogen phosphate Na_2_HPO_4_ (0.01 mol L^-1^), NaCl (0.15 mol L^-1^), NP-40 (1 %), sodium dodecyl sulfate (0.01 %) and Ethylenediamine tetraacetic acid (2 mmol L^-1^), pH 7.2) using a ball mill (Sartorius, Goettingen, Germany). The TP concentration was determined by means of a BCA assay kit (Thermo Fisher Scientific, Waltham, USA). 

For the label-free analysis, 20 µg protein tissue lysate was diluted 1:1 with 2x LDS buffer (NuPAGE™, Thermo Fisher). Samples were then denatured for 5 min at 90 °C. Protein from tissue lysate were run into 4-20 % gel (Novex™, Thermo Fisher) for about 1 cm (15 min and 100 V, 32 mA). The gel was then stained for 40 min at RT with instant staining buffer (GelCode™, Thermo Fisher). Protein bands were excised and subjected to tryptic digestion using an enzyme:protein ratio of 1:15 (Worthington-Biochem, Lakewood, USA) overnight at 37 °C. The resulting peptides were then extracted after 15 min ice sonication using 20 µl 50 % acetonitrile in 0.1 % trifluoracetic acid (TFA). The extraction cycle was repeated twice. The samples were then dried via vacuum centrifugation and dissolved in 0.1 % formic acid (FA). The peptide concentration was then determined via NanoDrop™ (Thermo Fisher Scientific, Waltham, USA). All samples were processed in triplicates.

For tissue-based verification by means of IA-LC-MS/MS analysis, lysates of 150 mg flash frozen kidney cortex sample(s), TEA buffer and lysis buffer were mixed and incubated at 99 °C for 5 min. Samples were subsequently reduced by tris(2-carboxyethyl) phosphine (TCEP, Carl Roth, Karlsruhe, Germany). After cooling, 2-Iodoacetamide (IAA, Sigma-Aldrich) was added. Samples were incubated for 30 min at room temperature. For proteolysis, trypsin (Worthington-Biochem, Lakewood, USA) was then added at a 1:20 ratio (trypsin:protein). Samples were incubated at 37 °C for 16 h. The reaction was stopped by heating up to 99 °C for 5 min and the subsequent addition of phenylmethylsulfonyl fluoride (PMSF, Roche Diagnostics). All samples were prepared in triplicate.

Urine samples were proteolytically fragmented in a 96-well microtiter plate. 50 µl volume urine was diluted with 40 µl digestion buffer (100 mM triethylammonium bicarbonate + 0.5 % octyl-beta-glucoside). After protein denaturation (99 °C, 5 min), 10 µl 100 mM ammonium bicarbonate was added and samples were reduced by TCEP (550 mM, 56 °C, 5 min), alkylated by IAA (1200 mM, 23 °C, 20 min.) and digested with trypsin at 37 °C for 2 h (250 ng total trypsin amount). The digestion was stopped by adding 210 mM PMSF.

### Proteomic label-free analysis

#### LC-MS/MS analysis

The 27 tissue samples were analyzed in randomized order by alternating the control and the treated samples. In addition, three pools of all samples were analyzed, at the beginning, in the middle and at the end of the analysis period. LC-MS/MS analysis was performed using an Q Exactive™ Plus Hybrid Quadrupole-Orbitrap™ Mass Spectrometer coupled to an Ultimate™ 3000 RSLCnano System (Thermo Scientific, Bremen, Germany). 500 ng of peptides was preconcentrated for 5 minutes on a trap column (Acclaim PepMap 100, 300 μm × 5 mm, C18, 5 μm, 100 Å, Thermo Fisher Scientific) at a flow rate of 20 μL min^-1^ (2 % ACN, 0.05 % TFA) and subsequently separated on an analytical column (Acclaim PepMap RSLC, 75 μm × 50 cm, nano Viper, C18, 2 μm, 100 Å, Thermo Fisher Scientific). Buffer A (0.1 % FA) and buffer B (0.1 % FA, 80 % ACN) were used to elute the peptides from the analytical column using a gradient ranging from 4 % to 45 % buffer B at a flow rate of 400 nL min^-1^ over 92 minutes (column oven temperature 55 °C). The MS was operated in a data dependent-mode. Full MS scan spectra were acquired in the orbitrap analyzer at a resolution of 70,000 in the profile mode. The mass range was set as 350-2000 m/z. The 20 most abundant precursor ions in the MS scan were selected for subsequent MS/MS analysis following precursor fragmentation by collision-induced dissociation (CID). The mass window for precursor isolation was 1.6 m/z. We used charge screening and only precursors having charge states of +2, +3 and +4 were fragmented by means of normalized collision energy (CE) set at 27. Dynamic exclusion was set at 50 s.

#### Protein identification and quantification

Ion intensity-based label-free quantification was performed using Progenesis QI (ver. 2.0.5387.52102, Nonlinear Dynamics Ltd., Newcastle upon Tyne, UK). The retention time was aligned to correct any potential retention time shift. pooled sample measurements were used as a reference. Only features with charges from +2 to +5 were included. The combined MSMS spectra was then exported; and a search was done in the UniProt database (Release 2020) via Mascot (ver. 2.3.2) (Matrix Sciences Ltd., London, UK) and SEQUEST was performed using the Proteome Discoverer (version 2.1) software (Thermo Fisher Scientific, Rockford, IL, USA). The search parameters were as follows: 5 ppm precursor mass tolerance, 0.4 Da fragment mass tolerance, trypsin as the enzyme; and two missed cleavage sites were allowed, with amino acid oxidation (i.e., H (histidine), M (methionine) and W (tryptophan)) set as the dynamic modification and carbamidomethyl for static modification for cysteine (C). The percolator program implemented in Proteome Discoverer was used to calculate the false discovery rate (FDR) of the identified peptides and only peptides with FDR < 1 % were considered. The search results deriving from the Proteome Discoverer were imported into Progenesis QI to combine feature quantification with peptide identification. Only unique peptides for a corresponding protein were used for quantification. The protein grouping option integrated in Progenesis QI was disabled. For protein quantification, protein abundance was extrapolated from the sum of unique normalized peptide ion abundances corresponding to that protein. Only non-conflicting peptides were considered for the relative quantification.

#### Gene ontology analysis

Comprehensive gene enrichment analysis was done, based on WikiPathways 2019 (Slenter et al., 2018[33]) in the web-based server Enrichr database. The ranking of enriched pathways was based on the FDR corrected *p-value* presented by Enrichr (Kuleshov et al., 2016[23]).

### Immunoaffinity liquid chromatography tandem mass spectrometry assay

#### IA-LC-MS/MS Assay development

To confirm differentially regulated protein biomarker candidates, we developed quantitative IA-LC-MS/MS assay using Triple-X-Proteomics (TXP) antibodies (Hoeppe et al., 2011[19]). Polyclonal antibodies specific for the c-terminus of four amino acids of tryptic peptides (TXP-abs) were generated as previously described (Hoeppe et al., 2011[19]). Peptides for the selected proteins were synthesized (Intavis Ag, Tuebingen, Germany) as stable isotope-labeled analog (C-terminal lysine or arginine was ^13^C/^15^N-labeled) as an internal standard peptide (SI-peptide). Additionally, synthetic versions of the endogenous peptides (EN peptides) were synthesized. All peptides were purchased in purified quality of approximately < 99 %. The TXP Abs and their corresponding c-terminal epitopes are summarized in Table 1(Tab. 1). In tissue analysis, due to beads-Ab capacity limit, AMBP, CST3, RBP4, OPN and CLU were analyzed in one assay. KIM-1 was analyzed separately as it needed a high amount of TXP antibodies to achieve higher protein recovery. Serpin A5 (SERPINA5), fatty acid-binding protein FABP4 and Poly (RC) binding protein 1 (PCBP1) were analyzed in a separate assay.

#### IA-LC-MS/MS analysis

40 µg proteolyzed tissue lysate or 50 µl digested urine samples were mixed with TXP antibodies in a 96-well PCR microtiter plate. The immunoprecipitation process was described previously (Gautier et al., 2016[15]; Weiss et al., 2018[41]). After eluting peptides in 20 µl 1 % FA, one-quarter of the peptides was preconcentrated for 0.25 min. on the trap column Acclaim (0.3 mm I.D. x 5 mm, 5 µm, Thermo Fisher Scientific) at a flow rate of 120 µL/min (2 % ACN and 0.05 % TFA). The peptides were then separated over 5 min by an Acclaim PepMap RSLC C18 (75 µm I.D. x 150 mm, 2 µm, Thermo Fisher Scientific) using a gradient (4 % to 35 % over 4.5 min) of 1 µL/min at 55 °C. The MS was operated in parallel reaction monitoring mode (PRM). The resolution was set to 70,000 and AGC target to 2E5, with maximum injection time 120 mins and a 1.5 m/z mass isolation window. Collision energy was set to 25 for all peptides. The maximum number of peptides per scan event was set to two. 

#### Protein quantification in tissue by IA-LC-MS/MS

PRM data were processed using Skyline 4.1 (MacLean et al., 2010[26]). The peaks were automatically integrated and the Savitzky-Golay smoothing algorithm was applied. Peak integration was manually checked. The peak area ratios for endogenous/SI-peptide were exported. The abundance of the peptides in the tissue samples was compared with the SI-peptide, converted to femtomoles (fmol), and normalized using the analyzed protein amount (µg). 

In the control group, baseline signals for KIM-1 and FABP4 proteins in the proteolytic tissue lysate were below the detection limit. Yet, KIM-1 and FABP4 proteins were detected in tobramycin-treated dogs with moderate tubular damage (example for KIM-1 signal is illustrated in Supplementary information, Figure 1). To impute the missing values, we used half the smallest concentration observed in the dataset for each protein. LLOQ was not defined at this stage of the assay. The rationale for imputing the missing values is to estimate what the undetectable levels might have been, without the biases that could result from assigning them a zero or excluding them (Donders et al., 2006[11]). We believe that the missing data we experienced here are data below the detection limit and therefore they are missing not at random (MNAR). Wei et al. showed that MNAR omics data are best imputed via quantile regression imputation (QRILC) or half minimum observed concentration with a slight advantage for QRILC (Wei et al., 2018[40]). Yet, the half minimum method was applied here as it is straight forward, transparent, easier to interpret and does not need computational work. 

#### Urinary protein quantification by IA-LC-MS/MS

PRM data analysis was performed as mentioned earlier in tissue PRM data analysis. The peak area ratios for endogenous and SI-peptides were exported. Urinary protein quantification was calculated based on single point calibration (Gerber et al., 2003[16]). LLOQs were determined by the determination of intra- and inter-assay precision analysis (n=4) of a peptide standard curve showing a precision of < 25 % and a total error < 40 % for urinary (UR) AMBP, UR CST3, UR RBP4, UR OPN, UR CLU, and UR KIM-1. The LLOQs ranged between 5 ng/mL for RBP4 to 61 ng/mL for CST3. Serpin A5 and FABP4 were not included in this assay development workflow but were screened separately in the urine samples. Results below the LLOQ were replaced with half the detection limit (LLOQ/2). All generated data were normalized to urinary creatinine. 

### Statistical analysis

Label-free protein abundance of the technical triplicates (control dogs; n=3 and tobramycin-treated dogs; n=6) were used to calculate the *p-value* of Student's t test (independent samples, two-samples t-test, assuming equal means). For later analysis FDR-corrected *p*-values generated via Progenesis QI (*P_FDR_-value*) were used to evaluate the significant changes between the different treatment groups.

## Results

### Histopathology showed tobramycin-induced renal injury 

Table 2(Tab. 2) provides a summary of the microscopic kidney injury scores and related characterization for all dogs. There were no gross renal pathological findings. Tobramycin-induced histopathologic changes included bilateral, multifocal, minimal to moderate proximal tubular necrosis, minimal to moderate intratubular granular casts, and minimal to slight/mild interstitial acute inflammation (n=6; Figure 2B(Fig. 2)). Minimal to slight/mild hyaline casts were also identified in the tobramycin-treated dogs. There were no microscopic findings in the control (vehicle) group (n=3; Figure 2A(Fig. 2)). Notably, the proximal tubular injury was evident in absence of changes in routine blood-based clinical chemistry parameters, BIN and sCr.

### Differential proteome analysis showed several up-regulated and down-regulated proteins in response to the tobramycin-induced renal injury

**Figure 3:**
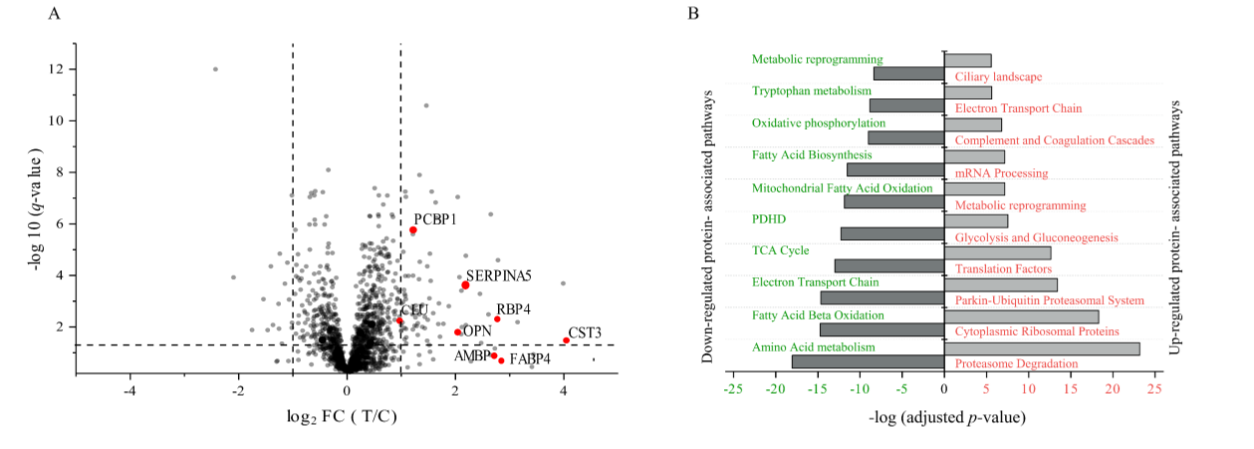
Male beagle dogs received once daily administration of tobramycin (60 mg/kg/day; n=6) or vehicle (saline; n=3) by intramuscular injection for 10 consecutive days; and necropsy on Day11. (A) volcano plot demonstrating the protein expression profiling between tobramycin-treated vs. control (vehicle)-treated dogs using label-free quantitative analysis. The dashed horizontal and vertical lines represent the threshold of statistical significance (*P_FDR _*< 0.05) and fold change criteria (FC > 2 or FC < -2), respectively. The highlighted dots in red represent the selected candidates for the multiplex IA-LC-MS/MS verification analysis. (B) Bar chart represents gene ontology-based classification of biological pathways. The bars visualize the enriched biological pathways among proteins with altered expression levels. The biological pathways were categorized based on the differential down-regulation (left) and up-regulation (right) proteins. Higher absolute values on the x-axis indicate greater statistical significance.

We sought to identify the underlying proteomic changes associated with proximal tubular injury in tobramycin-treated dogs. Additionally, we anticipated that this analysis would not only allow the discovery of novel potential preclinical kidney safety biomarker candidates in male beagle dogs after inducing tubular cellular damage but would also provide insights into the molecular mechanisms associated with DIKI. Representative tissue samples were collected at necropsy on Day 11 (Figure 1(Fig. 1)). The label-free proteomic analysis on kidney cortex tissue samples was performed in both tobramycin- and vehicle control-treated dogs. In total, 3019 proteins were quantified. Hence, 1731 proteins were detected and quantified with at least two unique peptides (Figure 3A(Fig. 3)). The entire data set is summarized in Supplementary data, Table S1. In the kidney cortices of the tobramycin-treated dogs, 77 proteins were significantly differentially up-regulated and 17 proteins were significantly differentially down-regulated at FC > 2 or FC < -2 and P_FDR_-value < 0.05, respectively (Figure 3A(Fig. 3)).

Gene ontology analysis revealed that down-regulated proteins (706 proteins) were associated with amino acid metabolism, fatty acid metabolism, beta-oxidation, oxidative stress, and carbohydrate metabolism (Figure 3B(Fig. 3)). There were 1022 differentially up-regulated putative proteins which were strongly associated with proteasome degradation and ribosomal proteins involved in translation (Figure 3B(Fig. 3)). Overall, these data highlight some underlying molecular mechanisms possibly associated with cell death likely related to the identified proximal tubular necrosis. 

### Biomarkers selection and verification in kidney cortex tissue via IA-LC-MS/MS confirmed tobramycin-induced changes in renal tissue injury and functional biomarkers

For verification of the proteomic results, three kidney function protein biomarker candidates (i.e., AMBP, CST3 and RBP4) and two kidney injury response protein biomarker candidates (i.e., OPN and CLU) were selected for further analysis. All five proteins, in the label-free analysis, were up-regulated in the tobramycin-treated dog kidney cortex tissue samples (FC > 2) (Supplementary data, Table S1). The selected candidates were previously published as preclinical biomarkers of tubular alterations in rats (Dieterle et al., 2010[10]; Harpur et al., 2011[18]; Vlasakova et al., 2014[38]). These proteins were selected with the aim of verifying their applicability as preclinical safety biomarkers in dogs. Additionally, we included KIM-1 as an additional protein for further analysis, although it was not identified in the label-free study. KIM-1 was proven to be an excellent biomarker for proximal tubular injury response in the rat, monkey, and humans (Gu et al., 2018[17]). Yet, little is known about this protein in dog preclinical studies. 

Furthermore, from the label-free analysis, three differentially up-regulated proteins PCBP1, Serpin A5 and FABP4 were selected for further verification analysis. PCBP1 was selected as it had previously been reported to be a target for proteasomal degradation (Zhang et al., 2017[43]), a pathway that was enriched in association with differentially up-regulated proteins. Serpin A5 was also selected based on its reported association with acute kidney injury (Vilander et al., 2017[37]). FABP4 protein was recently recommended as a potential novel rat preclinical safety biomarker of drug-induced kidney injury (Obajdin et al., 2018[30]), however, its performance to our knowledge has not yet been demonstrated in dogs.

According to the protein sequence database uniport, the annotation of the protein-coding and non-protein-coding gene sets has not yet been fully ascertained for dogs. Our selection of peptides for the verification analysis was thus based on sequence alignment with human, mouse, rat, and monkey proteins (data not shown). To ensure the right sequence as well as to verify the label-free data for the selected proteins, we investigated the selected protein biomarker candidates via IA-LC-MS/MS in the kidney cortex tissue (Table 1(Tab. 1)).

**Figure 4:**
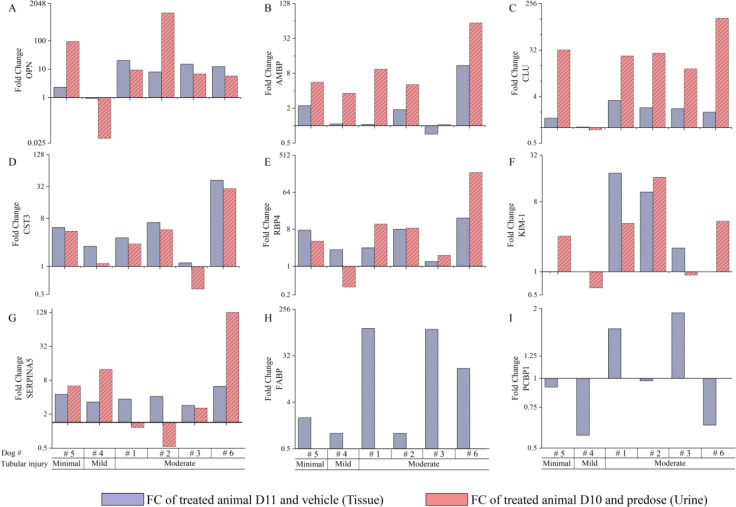
Relative quantification of drug induced kidney injury biomarker candidates in kidney tissue and urine samples in male beagle dogs after once daily administration of tobramycin (60 mg/kg/day; n=6) or vehicle (saline; n=3) by intramuscular injection for 10 consecutive days; and necropsy on Day11. Bar chart representing fold change in protein levels across individual animals (dogs: #1 to #6) with varying degrees of tobramycin-induced renal tubular injury. Dogs were grouped by the severity of renal tubular injury which were categorized as minimal, slight/mild, moderate and/or marked. The blue bars (dense pattern) represent the fold change in kidney tissues from the tobramycin-treated animals compared to the controls on Day11. The red bars (sparse pattern) represent the fold change in urine from the tobramycin-treated animals compared to their pre-dose levels at Day -4 on Day 10. For selected tissue and/or urine samples, protein analytes were not detected (i.e., < LLOQ) for selected dogs, including KIM-1 (i.e., all controls and tobramycin-treated animals #4 and #6) and FABP4 (i.e., all controls and tobramycin-treated animals #2 and #4) missing; therefore, values in tissue samples were imputed by using half the lowest concentration observed. The missing values in urine samples were imputed by using ½ LLOQ and normalized to concurrent UR CR concentrations.

As shown in Figure 4A to 4I(Fig. 4), the tissue-based verification analysis revealed that for the minimal to mild renal tubular injury, CST3, RBP4 tissue biomarkers' concentration increased up to 8-fold. Serpin A5 concentration increase was also observed but to a lesser extent (i.e., 4-fold). All other biomarkers did not show a notable change in minimal to mild renal tubular injury group. 

For the moderate renal injury, there was an increase in protein biomarker concentrations ranging from a 2-fold to >/= 100-fold increase, with FABP4 (dogs: #1, #3) and CST3 (dog #6) noted at the high end of the spectrum. On the other hand, KIM-1 (dogs: #1 and #2), AMBP (dog #6), OPN (dogs: #1, #2, #3 and #6), CST3 (dogs: #1 and #2) and RBP4 (dogs: #2 and #6) were shown to be in the mid-spectrum. Serpin A5, and CLU only showed minimum changes. As such, regardless of the magnitude of the renal injury, CST3 was the most consistent renal tissue biomarker in dogs. PCBP1 concentration did not show any changes following tobramycin-induced proximal tubular injury (Figure 4I(Fig. 4)).

Moreover, in Figure 4A to 4I(Fig. 4), correlative urine biomarker analysis on Day 10 showed that for the minimal to mild renal tobramycin-induced injury, the increased biomarker concentrations ranged from < 3 to >/= 95-fold with detectable changes in the levels of OPN and CLU at the high end of the spectrum (i.e., in dog #5). These findings are in contrast to the tissue biomarker findings mentioned earlier. Other biomarkers AMPB, RBP4 and CST3 were in the low point range. No increase was observed for Serpin A5 and KIM-1 in minimal to mild renal injury. 

For moderate renal tubular injury, the increase in biomarkers ranged from 3 to >/= 900-fold increases with most biomarkers hitting the high end of the spectrum in at least one animal (i.e., OPN in dog #2, AMPB, CLU, CST3, RBP4 and Serpin A5 in dog #6). Interestingly, CLU was consistently in the mid end of the fold change spectrum. Urinary KIM-1 concentration was increased in dogs #1, 2 and 6 while CST3 concentration increased (i.e., in dogs: #2, #5, and #6) at the low end of the fold change spectrum. Taken together, regardless of the magnitude of the renal injury, OPN, AMBP and CLU were the most sensitive and consistent urinary biomarkers in dogs using this technology.

### Temporal detailed analysis of urinary protein biomarker candidates using IA-LC-MS/MS showed tobramycin-induced changes 

**Figure 5:**
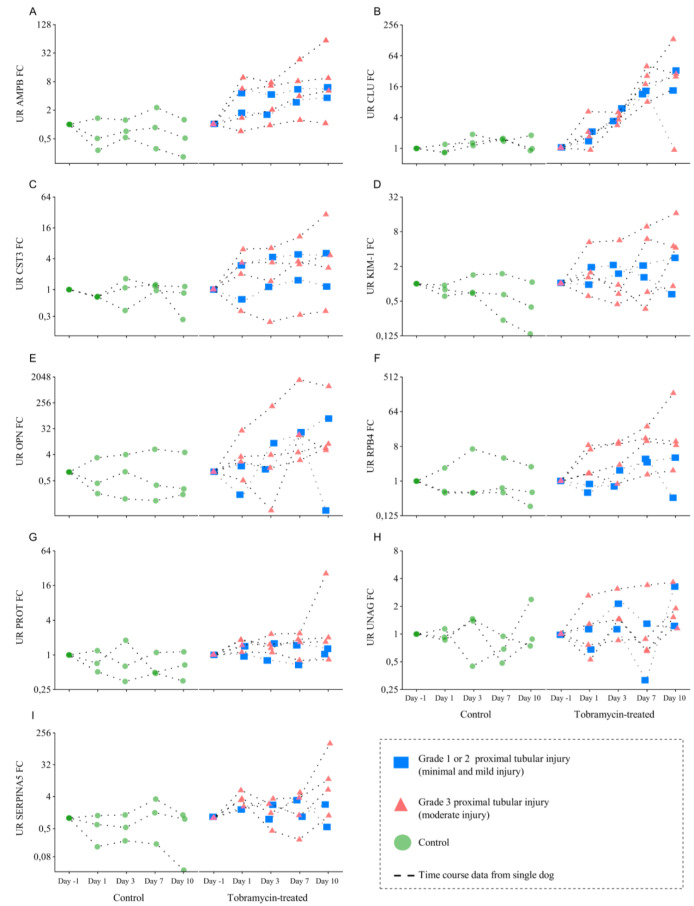
Time course of urinary kidney safety biomarker candidates in male beagle dogs after once daily administration of tobramycin (60 mg/kg/day; n=6) or vehicle (saline; n=3) by intramuscular injection for 10 consecutive days; and necropsy on Day11. The Scatter plot show UR AMBP (A), UR CLU (B), UR CST3 (C), UR KIM-1 (D), UR OPN (E), UR RBP4 (F), and UR Serpin A5 (I) fold change (FC) in control and Tobramycin-treated dogs. All analytes were measured with the developed multiplex IA-LC-MS/MS assay. NAG (H) and total protein (G) were determined using standard methods. The urinary biomarkers were normalized to urinary creatinine. Blue squares indicate tobramycin-treated dogs with Grade 1 or 2 proximal tubular injury (minimal and slight/mild injury), while red triangles represent dogs with grade 3 proximal tubular injury (moderate injury). Green circles denote control dogs. Dashed lines connect time course data from individual dogs, illustrating the progression of protein levels in response to treatment. Data < LLOQ were replaced by ½ LLOQ and normalized to uCr. The LLOQ for Serpin A5 was not determined.

Representative urine samples were collected from all animals on study at pre-treatment (Day -4) and pre-dosing (Days 1, 3, 8 and 10) and at necropsy on Day 11 (Figure 1(Fig. 1)). UR AMBP and UR CLU showed an early and sustained remarkable increase in the majority of the tobramycin treated animals, regardless of the magnitude of the renal tubular injury at histopathology (Figure 5A and 5B(Fig. 5)). This showed the sensitivity of these biomarkers in detecting early renal tubular injury in urine samples using this technology. The concentration of both proteins UR AMBP, and UR CLU continued to rise over days with the maximum fold change at Day 11 with 60-fold and 130-fold increase, respectively, when compared to the levels on Day -4 (i.e., days before tobramycin treatment).

On Day 2, UR CST3 elevation was seen in two animals with moderate proximal tubu-lar injury. UR CST3 increase was also observed in one treated dog with minimal and mild tubular injury but not in vehicle dogs (Figure 5C(Fig. 5)). UR OPN levels did not show a discernible increase in the treated dogs until Day 9 of treatment. Additionally, on Day 9, dogs #4, 5 and 6 demonstrated an approximate 19-fold increase, with dog #2 showing 1500-fold change (Figure 5(Fig. 5)) while in control animals, a 4-fold increase in OPN was observed only in one animal (i.e., dog #2).

UR RBP4 was elevated up to 8-fold in two treated dogs (#1 and #6) on Day 2 and its level crossed 190-fold in dog #6 at necropsy on Day 11. UR RBP4 elevation was seen in one control dog (dog #1) (Figure 5F(Fig. 5)). We cannot confirm the reason for this increase, but the consistent concentration of all other markers at baseline low level in this dog in addition to the normal histological finding, suggests that this observation is not due to a damage response.

On Day 9, UR KIM-1 minimal elevations were observed in 2 of the 4 animals with moderate proximal tubular injury. On Day 11, three dogs with moderate proximal tubular injury showed elevated levels of UR KIM-1 with the highest concentration recorded at 46 ng/mL in dog #1 (4-fold change) and 22 ng/mL in dog #2 (17-fold change). No increase in UR KIM-1 was detected in control dogs (Figure 5(Fig. 5)).

On Day 11, UR Serpin A5 concentrations increased in three treated dogs with moderate renal injury, with the peak change of 130-fold. However, no notable elevation was observed in the Serpin A5 levels in dogs suffering from minimal and mild tubular injury (Figure 5I(Fig. 5)). 

The concentration of UR FABP4 was below our detection limit and could not be measured in the urine samples. 

Throughout the duration of the study, treated animals did not exhibit an increase in UR NAG concentration which remained stable over the course of the experiment. Similar to UR NAG, the concentration of UR PROT remained consistent over the treatment days, showing no significant increase (Figure 5G and 5H(Fig. 5)).

Taken together, UR AMBP, UR CLU and UR CST, and to a lesser extent UR RBP4 and UR OPN were the most sensitive markers to early tubular injury using this assay platform. 

Supplementary data, Table S2 contains a comprehensive table of results, which includes the concentration of urinary biomarker candidates in ng/mL and their values normalized to urinary creatinine.

## Discussion

We investigated changes associated with tobramycin-induced proximal tubular damage in dogs at the proteome level. Our results indicate that strong enrichment of proteins involved in metabolic pathways, such as amino acid and fatty acid metabolism, beta-oxidation and carbohydrate metabolism are associated with differentially down-regulated proteins. Mice studies showed that impaired fatty acid oxidation causes tubular epithelial fibrosis by inducing apoptosis (Kang et al., 2015[21]), a pathway involved in epithelial cell death (Gandhi et al., 2014[14]). On the other hand, strong enrichment of proteasome degradation pathway was linked with differentially up-regulated proteins in tobramycin-treated dogs. The proteasome system is possibly implicated in renal injury (Debigare and Price 2003[8]) and plays a critical role in mediating inflammation (Meyer-Schwesinger, 2019[28]) presumably via proteasomal activity in apoptosis and necrosis (Ali and Mocarski, 2018[2]; Delgado et al., 2014[9]; Kretowski et al., 2015[22]). The differential down-regulation and up-regulation of proteins associated with fatty acid oxidation and proteasomal pathways, respectively, partly indicate the molecular mechanisms associated with dogs' proximal tubular injury.

Based on the label free proteome data we identified FABP4 and Serpin A5 as potential DIKI protein biomarker candidates in dogs. FABP4, has been previously studied for its association with DIKI in rat models (Obajdin et al., 2018[30]). Urinary FABP4 concentration was reported to be elevated after puromycin-induced glomerular damage. Similar observation was also reported after N-phenylanthranilic acid (NPAA)-induced necrosis in rats' kidney. However, inducing tubular injury via cisplatin had no effect on urinary FABP4 level (Obajdin et al., 2018[30]). In two other studies increased FABP4 protein abundance has been observed after cisplatin induced kidney damage in renal tubular cells (Li et al., 2021[24]; Tan et al., 2019[34]). We found that the FABP4 protein amount was drastically up-regulated in the kidney cortex after tobramycin-induced kidney damage as was identified in 4 out of 6 dogs (Figure 4(Fig. 4)). FABP4 was not detectable in the dog urine samples of our study (probably due to insufficient sensitivity of the assay), which hindered the full evaluation of FABP4 in our study. Overall, our findings along with others (Obajdin et al., 2018[30]) (Li et al., 2021[24]; Tan et al., 2019[34]) highlight the importance of FABP4 as a potential preclinical safety biomarker candidate and emphasize the need to understand the potential applications of FABP4 in the field of drug safety assessment. In the literature, there is no evidence that higher concentrations of Serpin A5 protein are associated with DIKI but previous proteomic data indicate that Serpin A5 is expressed in the kidney (Samaras et al., 2020[32]). Nonetheless, a genetic variant was associated with acute kidney injury (Vilander et al., 2017[37]). Additional weight of evidence for associating Serpin A5 with kidney injury in dog is required as further investigation would verify whether it is reabsorbed or rather only partially secreted, possibly due to physiological differences.

We developed a multiplex immunoassay for analyzing six urinary kidney injury biomarker candidates in a single experiment. Four candidates (UR KIM-1, UR CLU, UR AMBP and UR CST3) had previously been qualified for preclinical use by the FDA, EMA and PMDA (Dieterle et al., 2010[10]). Additionally, two proteins, OPN and RBP4, have been advocated for early DIKI detection (Vaidya et al., 2008[36]). To our knowledge, multiplexed sandwich immunoassays for the analysis of samples from rodents and humans have been established, but this is the first multiplex assay allowing quantification of all six biomarker candidates in kidney tissues and urine derived from dogs in a single analysis (3 functional biomarker candidates AMBP, CST3 and RBP4 and 3 damage response biomarker candidates OPN, CLU and KIM-1).

For the tubular dysfunction markers, UR AMPB concentration was mostly increased in all moderate proximal tubular injury and to less extent in mild and minimal injuries. AMBP is synthesized in the liver and its free form is filtered by the glomerulus and it is normally reabsorbed by proximal tubule cells (Vaidya et al., 2008[36]). Previously, AMBP was found to be a promising marker for proximal tubular damage in dogs with chronic kidney disease (Cobrin et al., 2013[6]). Although UR AMBP is a promising marker of tubular damage, its application as a single DIKI marker is limited since its level responds to various conditions such as liver diseases (Penders and Delanghe, 2004[31]). The combination of UR AMBP with other DIKI protein biomarkers in a panel would be useful.

The other two small-molecular-weight functional protein biomarker candidates UR CST3 and UR RBP4 also exhibited a distinct response correlated with severe tubular damage. However, their response was not as pronounced in minimal and mild tubular damage, indicating kidney reabsorption function. 

In line with the data presented here, a previous preclinical tenofovir dog toxicological study showed 100-fold UR CST3 increase after tenofovir-induced tubular damage (Gu et al., 2018[17]). Others also recommended UR CST3 and UR RBP4 as glomerular and tubular biomarkers (Christensen et al., 1999[5]). Our findings along with Vlasakova and colleagues (2014[38]) indicated that tubular dysfunction markers cannot be applied as individual biomarkers. However, in a DIKI protein biomarker panel, the markers could provide further evidence of proximal tubular damage that may not be ascertained by other DIKI biomarkers. 

In case of the investigated three tissue injury response markers, CLU, KIM-1 and OPN we found that UR CLU demonstrated the best damage response curve among all investigated biomarker protein candidates including UR NAG and UR PROT. This observation corroborates previous reports (McDuffie et al., 2016[27]; Zhou et al., 2014[45]) and underscores the importance of UR CLU in detecting tubular injury in canine subjects.

Urinary KIM-1 has been qualified as a urinary renal biomarker for proximal tubular injury in preclinical studies (Dieterle et al., 2010[10]). Here, in dogs, elevated UR KIM-1 levels were detected on Day 10 in correlation with moderate proximal tubular injury (Figure 5D(Fig. 5)). Yet, UR KIM-1 increase was not comparable to what we observed in the kidney cortex. Very similar findings were recently reported by Adedeji et al., in amphotericin b-induced kidney injury in dogs' study (Adedeji et al., 2023[1]). KIM-1 is an excellent biomarker for monitoring renal injury in rats (Vlasakova et al., 2014[38]), non-human primates (Gu et al., 2018[17]) and humans (Vaidya et al., 2010[36]). Typically, KIM-1 protein overexpresses in response to enhanced apoptosis and necrosis, as these processes mediate the engulfment of apoptotic and necrotic debris of injured proximal tubule cells (Ichimura et al., 2008[20]). It was proposed that the extracellular region of KIM-1 is cleaved in a metalloproteinase and mitogen-activated protein kinase (MAPK) pathway-dependent manner generating a soluble urinary fragment that can be detected in urine (Bailly et al., 2002[3]; Zhang et al., 2007[44]). To date, it is not fully understood how MAPK and metalloproteinase pathways respond to drug-induced kidney damage and to what extent these pathways are activated in dogs. Hence, the role of KIM-1 in dogs also necessitated further investigation. We also cannot rule out the possibility that our results were masked by post-translational modifications preventing release of the targeted peptide during digestion or detection by MS. While experiments with recombinant protein refute this hypothesis, it still cannot be entirely excluded.

We observed UR OPN response delay to proximal tubular injury (Figure 5E(Fig. 5)), a finding reported in tenofovir-treated dogs (Gu et al., 2018[17]). Gu et al. attributed this delay to the fact that higher expression levels of OPN were present in distal tubule cells than in proximal tubular cells. Upon tubular damage, OPN is released in urine after indirect distal tubules damage downstream to proximal tubule injury.

In summary, we used a label-free quantitative discovery proteomics to identify 94 proteins that were differentially regulated in response to DIKI. We found FABP4 and Serpin A5 to be elevated in tissue of dogs with drug-induced renal tubular injury. Nevertheless, further research needs to be done to ascertain the value of these two DIKI candidates in the urine of dogs and other species. We examined, by means of MS-based multiplex immunoassay, the temporal association of six biomarker protein candidates OPN, RBP4, CST3, CLU, AMBP and KIM-1 with tobramycin-induced renal injury in dog kidney tissue and urine. In the tissue analysis, KIM-1 and FABP4 showed a remarkable fold change after tobramycin treatment but not in urine. In urine, AMBP and CLU showed better performance in identifying proximal tubular injury than the OPN, RBP4, CST3 and KIM-1. The question of why KIM-1 does not perform in dogs as it does in rats and humans as a biomarker of drug safety after treatment with nephrotoxic agents remains unanswered and still requires further investigation. To our knowledge, this is the first multiplex technology for the detection of renal biomarkers in dogs. We believe the combination of these assays will help scientists gain insights into the molecular mechanisms associated with DIKI in dogs while using the renal biomarkers to monitor DIKI during toxicology studies.

## Declaration

### Acknowledgments

This material is based upon work supported by Critical Path Institute's (C-Path) Predictive Safety Testing Consortium (PSTC), Nephrotoxicity Working Group (NWG). The authors would like to acknowledge and thank the members of PSTC for their scientific, financial, and in-kind contributions that supported these research activities, as well as the input from US FDA and EMA scientists who served as advisors. 

### Conflict of interest statements 

The authors declare the following competing financial interest(s): H.P. and O.P. are shareholders of SIGNATOPE GmbH. SIGNATOPE offers assay development and service using MS-based immunoassay technology.

### Funding

This work was partly funded by the German Federal Ministry of Education and Research (grant FKZ 031B0395 to Thomas O. Joos, Wael Naboulsi, Hannes Planatscher, Oliver Poetz, Felix F. Schmidt, and Andreas Steinhilber).

### Author contributions

Adedeji, A. O., McDuffie, J. E., Naboulsi, W., Planatscher H and. Poetz, O. contributed to conception, design, data analysis and interpretation, drafted and critically revised the manuscript. Schmidt, F.F., Steinhilber, A., contributed to data acquisition, analysis and critically revised the manuscript. Joos, T.O., drafted and critically revised the manuscript. All authors gave final approval and agreed to be accountable for all aspects of work ensuring integrity and accuracy.

## Supplementary Material

Supplementary information

Supplementary data

## Figures and Tables

**Table 1:**
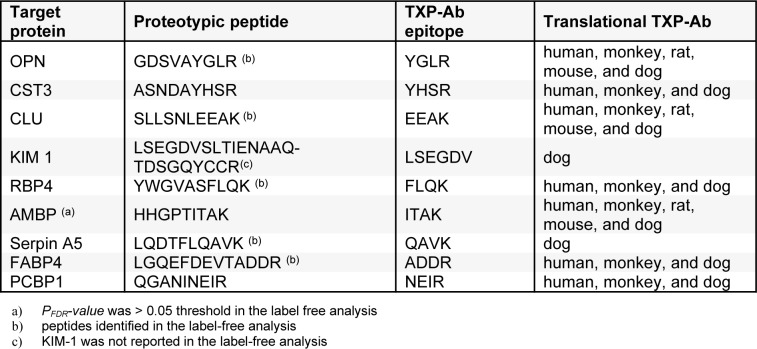
List of TXP antibodies and peptide sequences of the targeted protein biomarker candidates

**Table 2:**
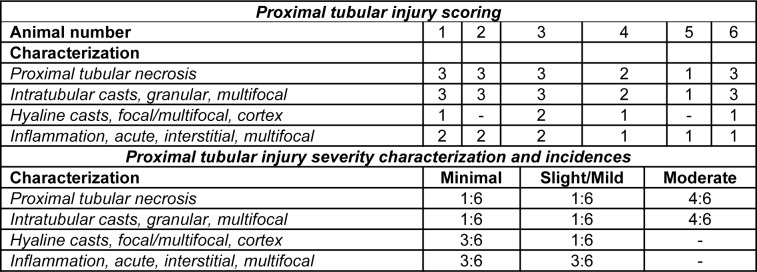
Proximal tubular injury scoring, characterization, and incidences for male beagle dogs (n=6) after once daily administration of tobramycin at 60 mg/kg/day by intramuscular injection for ten consecutive days. Data represent microscopic kidney (left and right) lesions identified from humanely euthanized animals at the scheduled necropsy on study Day 11. Histopathological characterization was performed using a semi-quantitative scale in which the percentages of affected proximal cortical tubules showing necrosis, intratubular cast, hyaline cast(s), or acute inflammation were assigned a score: Grade 1 = minimal; Grade 2 = slight/mild; Grade 3 = moderate; and Grade 4 = marked. There were no microscopic findings in the control (vehicle) group dogs (n=3; data not shown).
