# Supplementary material for: Immunoaffinity proteomics for kidney injury biomarkers in male beagle dogs
Source: EXCLI J. 2024 Jan 15;23:180–97. doi: 10.17179/excli2023-6621 (PMC10938254; doi:10.17179/excli2023-6621)
Supplement: Supplementary information [file EXCLI-23-180-s-001.pdf]

EXCLI Journal 2024;23:180-197 – ISSN 1611-2156
Received: September 20, 2023, accepted: January 15, 2024, published: February 05, 2024

Original article:

IMMUNOAFFINITY PROTEOMICS FOR KIDNEY INJURY BIOMARKERS IN MALE BEAGLE DOGS

Wael Naboulsi[1], Hannes Planatscher[1], Felix F. Schmidt[1], Andreas Steinhilber[1], Thomas O. Joos[1,2], Adeyemi O. Adedeji[3], J. Eric McDuffie[4,5], Oliver Poetz[1,2]

[1] Signatope GmbH, Reutlingen, Germany
[2] NMI Natural and Medical Sciences Institute at the University of Tuebingen, Reutlingen, Germany
[3] Department of Safety Assessment, Genentech, A Member of the Roche Group, South San Francisco, CA, USA
[4] Janssen Pharmaceutical Research & Development LLC, San Diego, California, USA
[5] Neurocrine Biosciences, Inc., San Diego, California, USA

* **Corresponding author:** Oliver Poetz, Signatope GmbH, Reutlingen, Germany, E-mail: poetz@signatope.com

https://dx.doi.org/10.17179/excli2023-6621

This is an Open Access article distributed under the terms of the Creative Commons Attribution License (http://creativecommons.org/licenses/by/4.0/).

EXCLI Journal 2024;23:180-197 – ISSN 1611-2156
Received: September 20, 2023, accepted: January 15, 2024, published: February 05, 2024

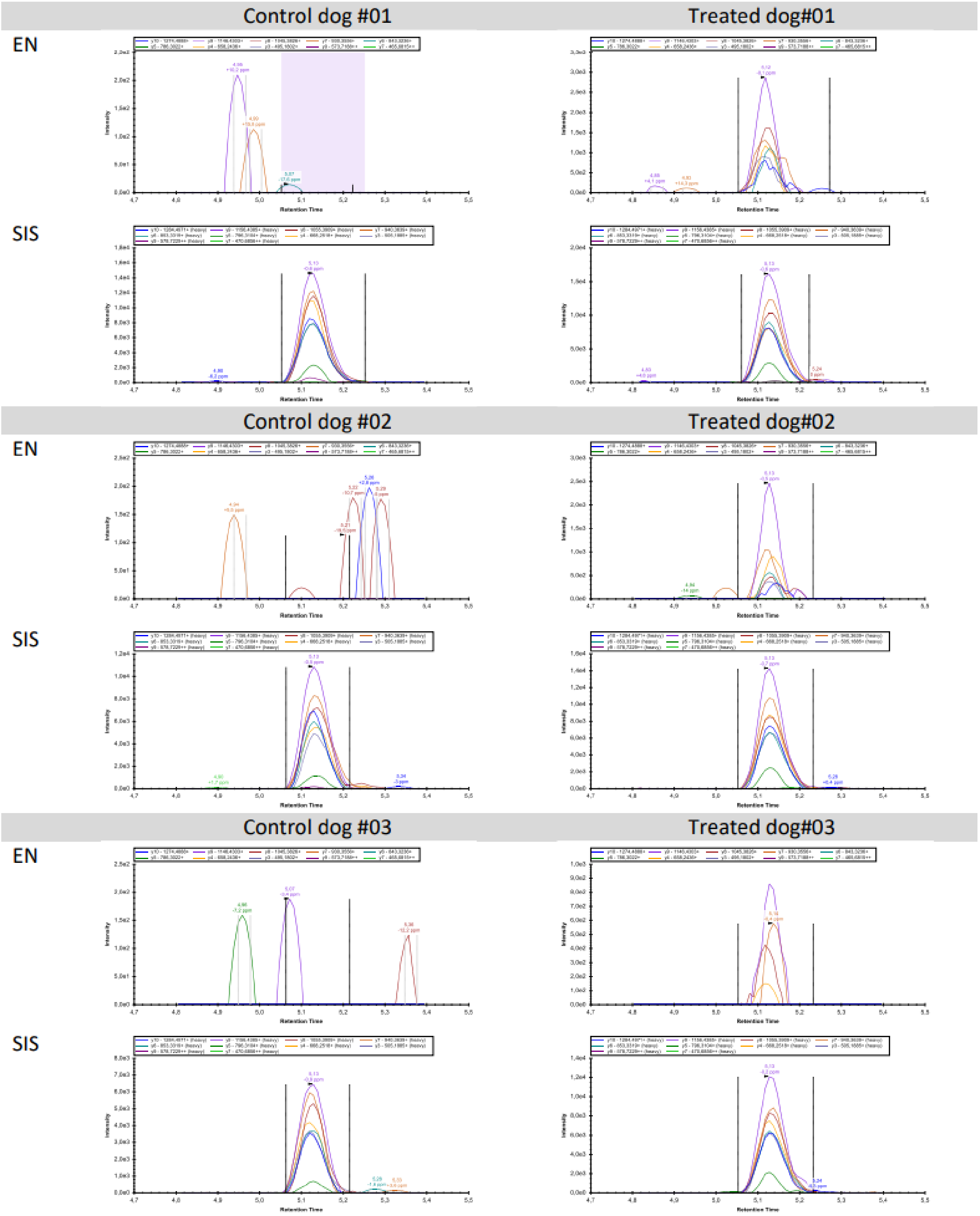

Supplementary Figure 1: This figure illustrates the base peak signal of KIM-1 in three control dogs and three treated dogs. The figure demonstrates a weak endogenous signal (EN) of KIM-1 in control dogs, while a more distinct signal is observed in treated dogs. The signal from the stable isotope-labeled standard (SIS) is comparable in both control and teated cases. All three treated animals #01, #02 and #03 had moderate tubular injury.
